# Dynamic changes occur in the cell wall composition and related enzyme activities during flower development in *Rosa damascena*

**DOI:** 10.3389/fpls.2023.1120098

**Published:** 2023-07-31

**Authors:** Sercan Önder, Muhammet Tonguç, Damla Önder, Sabri Erbaş, Murat Mutlucan

**Affiliations:** ^1^ Department of Agricultural Biotechnology, Faculty of Agriculture, Isparta University of Applied Sciences, Isparta, Türkiye; ^2^ Department of Biology, Faculty of Arts and Sciences, Suleyman Demirel University, Isparta, Türkiye; ^3^ Department of Field Crops, Faculty of Agriculture, Isparta University of Applied Sciences, Isparta, Türkiye

**Keywords:** cell wall, cellulose, enzyme activity, oil-bearing rose, pectin, petal

## Abstract

The flowering period of oil-bearing rose is short and many physiological processes occur during flower development. Changes in the cell wall composition and associated enzyme activities are important as they allow cells to divide, differentiate and grow. In the present study, changes in seven cell wall components and six cell wall-related enzyme activities at five flower development stages were investigated and the relationships between these parameters and flowering were examined. Ash content did not change between stages I to II but decreased at later stages. Neutral detergent fiber (NDF), acid detergent fiber (ADF) and hemicellulose contents increased after stage I but did not change significantly at the other developmental periods. Total pectin content increased throughout flower development. An “increase–decrease” trend was observed in total cellulose content and a “decrease–increase” pattern in uronic acid content. The activities of both glycosidases (β-galactosidase, β-glucosidase and endoglucanase) and pectinases (pectin lyase, pectin methyl esterase and polygalacturonase) increased until stage IV and decreased significantly at stage V of flower development. Correlation analysis revealed 14 positive and one negative correlation with the studied parameters. Cell wall enzymes showed positive correlations with each other. Principal component analysis (PCA) showed that ADF, NDF and cellulose content were significantly altered at stage II of flower development, and significant changes occurred in all cell wall enzyme activities between stages III and V. Overall, blooming is correlated closely with increased pectin and decreased cellulose contents, and changes in cell wall glucosidase and pectin hydrolysis enzyme activities. These results show that cell wall modifying enzymes are part of the flower development process in oil-bearing rose. Therefore, remodeling of cell wall components in petals is a process of flower development.

## 1 Introduction

Oil-bearing rose (*Rosa damascena*), locally known as Isparta gülü, is a member of the *Rosaceae* family and is an important essential oil-producing crop. It is highly valued for its essential oil, which is used in the perfumery, pharmaceutical, food and cosmetic industries. Oil-bearing rose has high economic value and is cultivated commercially in Türkiye, Bulgaria and Iran for its flowers, which are used for the production of rose oil, absolute and concrete. A high demand for good quality rose oil by different industries has caused an increase in oil-bearing rose cultivation and rose oil production. The blooming of the oil-bearing rose takes place once a year and continues for 25–30 days during May and June. Flowering from the bud stage to full blooming takes approximately 11–13 days, and the flower production rate reaches its height in early June (Baydar et al., 2013; Önder et al., 2022).

Flowering is an important aspect of the angiosperm life cycle allowing reproduction to take place. During flower development, significant changes occur in the polysaccharide levels and composition, color development, and production of primary and secondary metabolites within oil-bearing rose petals (Önder et al., 2022; Önder et al., 2023). The plant cell wall is a strong network of fibrils that are arranged to give each cell a consistent shape and a complex structure composed of a pectin matrix, proteins, tissue/organ-bound cellulosic microfibers and neutral non-cellulosic polysaccharides (McCann and Roberts, 1991; Carpita and Gibeaut, 1993). The plant cell wall shows great diversity in different species, cell types and even in subcellular cell wall domains (Derbyshire et al., 2007; Pelletier et al., 2010; Wolf et al., 2012). Despite the apparent diversity, pectins, hemicellulose, and cellulose form the main groups of polysaccharides in plant cell walls (Seymour and Gross, 1996; Brummell and Harpster, 2001; Yap et al., 2008). Pectins are structurally complex plant cell wall polysaccharides and have important roles in cell–cell adhesion. Although the mechanisms that regulate cell wall polysaccharides during flower development are not fully understood, information regarding changes in components, such as pectin, cellulose and uronic acid, has been obtained from different studies (O’Donoghue et al., 2002; Yap et al., 2008; Yang et al., 2021).

Changes in cell wall composition and structures of flowers occur due to the coordinated or interdependent action of a number of cell wall modifying enzymes. Cell wall-associated enzymes play important roles in cell wall development, remodeling and degradation during flower development (Yap et al., 2008; Zhang et al., 2019; Yang et al., 2021). Pectinases, such as polygalacturonase, pectin methyl esterase and pectin lyase, involved in these biochemical pathways, are among the enzymes that act on the pectin fractions in the cell wall. Pectin methyl esterase catalyzes pectin demethylation, breaking down calcium linkages in the acidic polysaccharide chain, thereby producing a substrate for polygalacturonase as well as cell separation for cell expansion (Brummell et al., 2004). During flower development, polygalacturonase affects pectin degradation and cell wall expansion by hydrolyzing pectic acid along with the main chain of polygalacturonic acid (Hadfeid and Bennet, 1998; Zhang et al., 2019). Pectin lyases degrade pectin polymers through a β-elimination mechanism yielding oligogalacturonides (Cao, 2012). Glucosidase enzymes, such as β-galactosidase, β-glucosidase and endoglucanase act on different carbohydrate substrates. β-galactosidase is the main glycosidase enzyme that removes the galactosyl component from the cell wall (Tateishi, 2008). β-glucosidase breaks β-glycosidic bonds, thereby breaking down polysaccharides and making sugars available (Opassiri et al., 2003; Mohapatra et al., 2010). Endoglucanase is an endo-hydrolase that acts on glucose polymers by cleaving glycosidic bonds. Its target in plant cell walls can be xyloglucans or mixed linkage glucans (Jara et al., 2019). The activities of the cell wall-related enzymes change during flower development, and these changes may cause major changes in petals (Panavas et al., 1998; Yap et al., 2008). In addition, the reduction of cell wall-related enzyme activities significantly delays the processes leading to senescence (Panavas et al., 1998). Any of these enzymes alone is not sufficient to cause changes in cell walls, but several isoforms of these enzymes and post-transcriptional regulatory mechanisms affect the overall process (Goulao et al., 2007).

Flower development is associated with the accumulation, solubilization and depolymerization of cell wall components, and the activities of different cell wall-related enzymes in flower development have been reported (Yap et al., 2008; Yang et al., 2021). However, the reported changes during flower development may be due to different cell wall modification mechanisms in different species (O’Donoghue et al., 2002; Yap et al., 2008), therefore, flowering plants need to be investigated individually. In addition, the flowers of various plants have commercial, industrial and aesthetic values and should be studied separately. To date, limited data are available on cell wall compounds and related enzyme activities in the petals of oil-bearing rose during flower development (Kumar et al., 2008). Therefore, the aim of the present study was to analyze changes in cell wall components and enzyme activities during flower development in oil-bearing rose petals. The findings can be used for a better understanding of the physiological processes associated with cell wall metabolism and regulation of flower development not only in oil-bearing rose but also in other flowering plant species.

## 2 Materials and methods

### 2.1 Plant material

Samples of oil-bearing rose (*R. damascena* Mill. var. *trigintipetale*) flowers at five stages of bud and flower development were collected from a commercial oil rose garden located in Ardıçlı village [latitude 37° 48’ 19.9” (N), longitude 30° 12’ 34.7” (E), 906 m altitude] in Isparta, Türkiye, between 6–8 AM on 22 May 2021. The oil-bearing rose plants were pruned in February and fertilized with 40 kg da^−1^ 10-18-12-20 (N-P-K-SO_3_) in winter. At the beginning of foliation, 1 L of fulvic acid, 2 kg of 10-52-10 (N-P-K) and 1 kg of micronutrient fertilizer (boron 1.3, copper 2.2, iron 7, manganese, molybdenum 0.03, zinc 4.3 w/w) were applied three times until flowering with drip irrigation system (Efecan et al., 2022).

**Figure 1:**
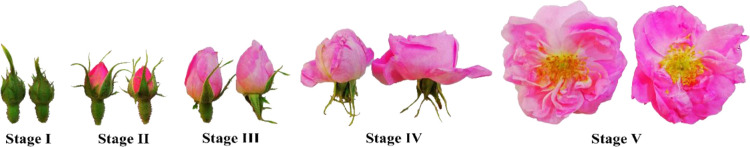
Five developmental stages of oil-bearing rose flowers used in the study: Stage I, buds with closed sepals; Stage II, sepals beginning to separate; Stage III, sepals separated and petals starting to enlarge; Stage IV, partially bloomed flowers; and Stage V, fully bloomed flowers.

The flower samples were harvested at the 5-flower stages as shown in **Figure 1**: Stage I, buds with closed sepals; Stage II, sepals beginning to separate; Stage III, sepals separated and petals starting to enlarge; Stage IV, partially bloomed flowers; and Stage V, fully bloomed flowers (Baydar et al., 2013; Önder et al., 2022). All samples were collected in three replicates, and delivered to the laboratory within an hour after the harvest and stored at −80°C for analysis. The information for mean temperature, total precipitation, and mean relative humidity of Isparta province obtained from the State Meteorological Service are given in **Table 1**.

**Table 1:** Long term (1929–2021) and monthly average temperatures (°C), monthly relative humidity (%) and monthly total rainfall (mm) for Isparta province recorded in 2021.

		Jan	Feb	Mar	Apr	May	Jun	Jul	Aug	Sep	Oct	Nov	Dec	Ave.
Temperature (°C)	2021	5.2	6.5	6.7	12.7	19.6	19.9	25.9	26.3	20.5	14.8	11.7	6.0	14.7
	1929–2021	1.8	3.0	6.0	10.7	15.5	19.9	23.4	23.3	18.9	13.4	7.9	3.6	12.3
Rainfall (mm)	2021	88.3	16.3	45.0	8.0	2.3	144.7	8.4	1.1	13.5	12.8	22.1	124.9	–
	1929–2021	81.0	67.0	58.7	51.6	56.4	35.5	15.8	14.1	18.5	38.1	44.6	87.1	–
Relative Humidity (%)	2021	77.8	62.7	62.2	54.6	42.2	58.8	39.6	34.3	47.6	53.7	61.9	76.8	56.0
	1929–2021	75.3	71.6	65.9	61.3	59.0	52.7	45.6	46.2	52.1	62.3	69.8	76.0	61.5

### 2.2 Determination of ash ratio of petals

To determine the ash content of the petals, 2 g of the sample was burned in a muffle furnace at 550°C for 6 h. Then, the samples were taken from the muffle furnace and cooled in the desiccator. After cooling, the samples were weighed, and the ash ratio was determined according to the following equation.


Ash Ratio (%) = ((Weight after combustion)/(Initial weight)) × 100

### 2.3 Determination of acid detergent fiber (ADF), neutral detergent fiber (NDF) and hemicellulose content of petals

ADF (Method 14) and NDF (Method 15) analysis were performed with ANKOM 220 Fiber Analyzer with chemical solutions purchased from ANKOM Technology (Macedon, NY, USA) following the manufacturer’s instructions (Ankom, 2017). For ADF, NDF and hemicellulose analysis, petals were separated from flowers and oven dried at 50°C until they reached a constant weight. For the determination of the ADF content of petals, 0.5 g of dried petal samples were placed into F57 bags, and the bags were sealed using heat treatment. The samples were placed in the fiber analyzer with three samples on each floor. An empty F57 bag was used for Blank (C1) and placed inside the device. Then 1.6 L acid detergent solution was added to samples and incubated in the device for 1 h. After the incubation, the solution was emptied and incubated with hot water at 90°C for 5 min. The washing was done with hot water and was repeated three times. After the washing step, the F57 bags were incubated in acetone for 3 min and dried in an oven at 105°C for 4 h. Afterward, the samples were cooled in a desiccator and then weighed (W3). The acid detergent fiber content in the petals was calculated according to the following equation.


$$\text{ADF }\left(\%\right)=\frac{(\text{W}3-\left(\text{W}1\times \text{C}1\right)\;\times \;100}{\text{W}2}$$

W1: Empty weight of the F57 bag, W2: Sample weight, W3: Weight after reaching room temperature in the desiccator and C1: Empty bag correction factor (weight after drying in the oven/initial empty bag weight).

NDF analysis was performed using the samples in F57 bags that remained after ADF analysis. F57 bags containing the samples were weighed and placed in the folded bag rack of the fiber analyzer with three samples on each floor. An empty F57 bag was used for Blank (C1) and placed inside the device as well. Then, 1.6 L of the neutral detergent solution was added and incubated in the device for 75 min. After the incubation, the solution inside was emptied, and hot water (90°C) and 4 mL of α-amylase solution were added and incubated for 3 min. The washing step with hot water was performed three times. After the washing step, the samples were incubated in acetone for 3 min and dried in an oven at 105°C for 4 h. Afterwards, the samples were allowed to cool in a desiccator and weighed (W3). The NDF content in petals was calculated according to the following equation.


$$\text{NDF }\left(\%\right)=\frac{(\text{W}3-\left(\text{W}1\times \text{C}1\right)\;\times \;100}{\text{W}2}$$

W1: Empty weight of the F57 bag, W2: Sample weight after ADF analysis, W3: Weight after reaching room temperature in the desiccator and C1: Empty bag correction factor (weight after drying in the oven/initial empty bag weight).

Hemicellulose content was calculated with the values obtained from ADF and NDF analysis using the following equation.


Hemicellulose (%)=NDF−ADF

### 2.4 Determination of pectin, cellulose and uronic acid content of petals

Cell wall extraction of the samples was carried out following Huber (1992) with some modifications. Petals (20 g) were homogenized in 80 mL of 95% cold-ethanol and incubated at −20°C for 24 h. The homogenate was centrifuged for 10 min at 8,000 × g at 4°C, and the supernatant was removed. Tris-HCl solution (100 mL) containing phenol (pH 7.5) was added to the pellet, and the resulting mixture was incubated for 45 min at room temperature. The homogenate was centrifuged again, and 100 mL of 80% cold ethanol was added to the pellet and incubated at −20°C for 2 h. The homogenate was centrifuged again, and the supernatant was removed. The remaining pellet was consecutively washed with 100 mL of 80% ethanol, 80% acetone and a chloroform:methanol (1:1) mixture, respectively, using glass fiber filters (GF/C, Whatman) under vacuum. To obtain crude cell wall material (ethanol-insoluble residue, EIS), the resulting residue was washed with 80% acetone, and EIS was obtained by drying the remaining residue at 40°C for 5 h. EIS samples were used to determine the pectin, uronic acid and cellulose contents of oil-bearing rose petals.

The amount of pectin in the EIS samples was determined according to the method of Ranganna (1977). Sodium hydroxide solution (100 mL, 0.05 N) was added to the EIS samples (100 mg) and incubated for 30 min to remove the ester groups in the pectin. After incubation, 2 mL of this solution was taken and the volume made up to 100 mL with distilled water. De-esterified solution (2 mL) was mixed with 1 mL carbazole solution (0.1%) and the mixture vortexed; then, 12 mL concentrated sulfuric acid was added slowly to the reaction mixture and incubated for 10 min at room temperature for color development. The absorbance of the samples was determined at 525 nm and known concentrations of galacturonic acid solution prepared with sodium hydroxide (0.05 N) were used to generate the standard curve.

The amount of uronic acid in EIS samples was determined according to the m-hydroxydiphenyl method (Melton and Smith, 2001). Concentrated sulfuric acid (3 mL) was added slowly to the EIS sample (5 mg) and incubated on ice for 5 min. After incubation, the sample volume was brought to 10 mL with deionized water, and the solution was incubated again on ice for 10 min and then centrifuged at 4,000 × g for 10 min. A total of 40 µL of 4 M sulfamic acid/potassium sulfamate (pH 1.6) solution and 2.4 mL of 75 mM sodium tetraborate solution were added to the supernatant and the reaction was vortexed. The reaction mixture was incubated in a boiling water bath for 20 min and then cooled in a cold water bath for 10 min. A total of 80 µL of m-hydroxydiphenyl solution (0.15%) was added to the reaction medium and incubated at room temperature for 10 min for color change. The absorbance of the samples was determined at 525 nm, and the absorbance value of the samples was subtracted from the control absorbance. Results were determined according to the generated galacturonic acid standard curve.

Cellulose content was determined with the Antron method (Updegraff, 1969). A total of 10 mL of acetic acid/nitric acid solution (10:1) was added to the EIS, and the samples were incubated in a boiling water bath for 30 min. The homogenate was centrifuged at 14,000 rpm at 4°C for 20 min, and after removing the supernatant, deionized water (20 mL) was added to the pellet and incubated for 20 min at room temperature. The mixture was centrifuged again, and the supernatant was removed. Sulfuric acid (67%) was added to the pellet and incubated on a shaker for 1 h. The mixture was then centrifuged again, and the cellulose content was determined from the resulting supernatant. Then, 10 mL of antron solution was mixed with 1 mL of the extract and incubated in a boiling water bath for 10 min. The absorbance of the samples was determined at 630 nm and cellulose concentrations of the samples were calculated from a standard curve prepared with cellulose.

### 2.5 Determination of activities of cell wall related enzymes in petals

For enzyme extraction, petals were frozen in liquid nitrogen and powdered with a mortar and pestle. A total of 10 mL of extraction solution (20 mM sodium phosphate, pH 7.5 and 1.5 M NaCl) was used per gram of fresh weight for the extraction of pectin methyl esterase, β-galactosidase, β-glucosidase, polygalacturonase, pectin lyase and endoglucanase. The mixture was incubated at 4°C for 30 min and the homogenate was centrifuged at 15,000 × g for 30 min at 4°C and enzyme activities were determined from the same supernatant for the enzymes listed above.

Pectin methyl esterase (EC 3.1.1.11) activity was determined according to Lohani et al. (2004). A 2 mL pectin solution (0.5% citrus pectin), 0.2 mL NaCl (0.15 M), 0.15 mL 0.01% bromothymol blue and 0.45 mL distilled water were added to the reaction tube. After the initial reading, the reaction was started by adding 0.2 mL of the enzyme extract, and the absorbance of the samples at 620 nm was determined at intervals of 30, 60, 90, 120, 150 and 180 seconds. The standard curve was constructed with 2 mL citrus pectin solution, 0.15 mL bromothymol blue and known concentrations of galacturonic acid (Hagerman and Austin, 1986). The specific enzyme activity was expressed as U mg^−1^ protein.

β-Galactosidase (EC 3.2.1.23) activity was determined following Borooah et al. (1961) and Kuby and Lardy (1953) with minor modifications. Distilled water (300 µL) was added onto 500 µL of substrate solution containing 0.01% Bovine serum albumin (BSA) and 2 mM o-Nitrophenyl β-D-Galactoside and incubated at 37°C for 10 min. Then, the reaction was started by adding 200 µL of enzyme extraction and incubated at 37°C for another 10 min. After incubation, 4 mL of 1 M sodium carbonate solution was added to stop the reaction and the absorbance value of the sample was determined at 405 nm. One unit of enzyme was defined as the amount of enzyme that hydrolyzes o-nitrophenol and D-galactose from 1 µmol of o-nitrophenyl β-D-galactoside per minute at a pH of 6.0 at 37°C. Results were expressed as U mg^−1^ protein.

β-Glucosidase (EC 3.2.1.21) activity was determined according to Gerardi et al. (2001) and Ross et al. (1993). A total of 4 mL of salicin substrate (1%) was added and incubated at 37°C for 5 min. The reaction was started by adding 1 mL of enzyme extract and incubated at 37°C for 10 min. After incubation, 1 mL of copper solution (16 mM copper sulfate, 1.3 M sodium sulfate, 0.226 M sodium carbonate, 0.19 M sodium bicarbonate and 43 mM sodium potassium tartrate) was added to the reaction mixture, and the mixture was incubated in a boiling water bath for 10 min. The reaction was cooled in a cold water bath and 1 mL of arsenate-molybdate solution (40 mM ammonium molybdate tetrahydrate, 19 mM sodium arsenate and 756 mM sulfuric acid) and 10 mL of deionized water were added. The absorbance of the samples was determined at 540 nm, and the specific enzyme activity was calculated using the glucose standard curve. One unit of enzyme was defined as the amount of enzyme that releases 1 μmol of glucose from salicin per minute at a pH of 5.0 at 37°C. Results were expressed as U mg^−1^ protein.

Polygalacturonase (EC 3.2.1.15) activity was determined with the 3,5-dinitrosalicylate (DNS) reagent method (Couri et al., 2013). The enzyme extract (0.1 mL) was mixed with 4 mL of 0.25% polygalacturonic acid solution and the solution was incubated at 35°C for 30 min. After incubation, the reaction was stopped by mixing 0.5 mL of the DNS reagent with 0.5 mL of the reaction mixture. The mixture was incubated in a boiling water bath for 5 min and cooled in a cold water. The absorbance of the sample was determined at 540 nm after adding 5.5 mL of deionized water. The specific enzyme activity was calculated using galacturonic acid as standard. One unit of enzyme was defined as the amount of enzyme that releases 1 μmol of galacturonic acid under standard assay conditions. Results were expressed as U mg^−1^ protein.

Pectin lyase (EC 4.2.2.10) activity was determined by mixing 2.5 mL of 0.5% apple pectin solution, 0.5 mL of 4 mM calcium chloride and 21 mL of 4 mM sodium acetate solution (pH 5.5) according to Couri et al. (2013). The mixture was incubated at 35°C for 10 min, and 1 mL of enzyme extract was added into the solution and incubated at 35°C for 15 min. The absorbance of the sample was determined at 235 nm and the specific enzyme activity was calculated using the standard curve prepared with uronic acid. One unit of enzyme was defined as the amount of enzyme that releases 1 μmol of unsaturated uronic acid per minute under standard assay conditions. Results were expressed as U mg^−1^ protein.

Endoglucanase (EC 3.2.1.4) activity was determined according to the DNS reagent method (Rahman et al., 2018). The enzyme extract and substrate solution (sodium carboxymethyl cellulose, CMC) were incubated at 50°C for 5 min, and 0.5 mL of 2% CMC solution was added to the enzyme extract and re-incubated at 50°C for 30 min. Then, 3 mL of DNS reagent was added, and the samples, blanks and glucose standards were incubated in a boiling water bath for 5 min. Finally, the reaction was cooled and 20 mL of deionized water was added. The absorbance of the samples was determined at 540 nm and the results were calculated with the use of a standard curve prepared with glucose and the results were expressed as U mg^−1^ protein.

The amount of protein in the enzyme extractions was determined according to the Bradford method (Bradford, 1976) and BSA was used to obtain the standard curve.

### 2.6 Data analysis

All biochemical analyses were performed in triplicate. The results were evaluated by analysis of variance (ANOVA) using SPSS Statistics 22.0 software (IBM, Armonk, NY, USA). The least significant differences (LSD, *P ≤* 0.05) test was used to distinguish the differences between the means. Pearson linear correlation analysis (Heatmap correlation) was calculated using OriginPro software (version 2021, OriginLab, Northampton, MA, USA) to show the relationship between the measured parameters. All values are expressed as mean ± standard deviation.

## 3 Results

**Figure 2:**
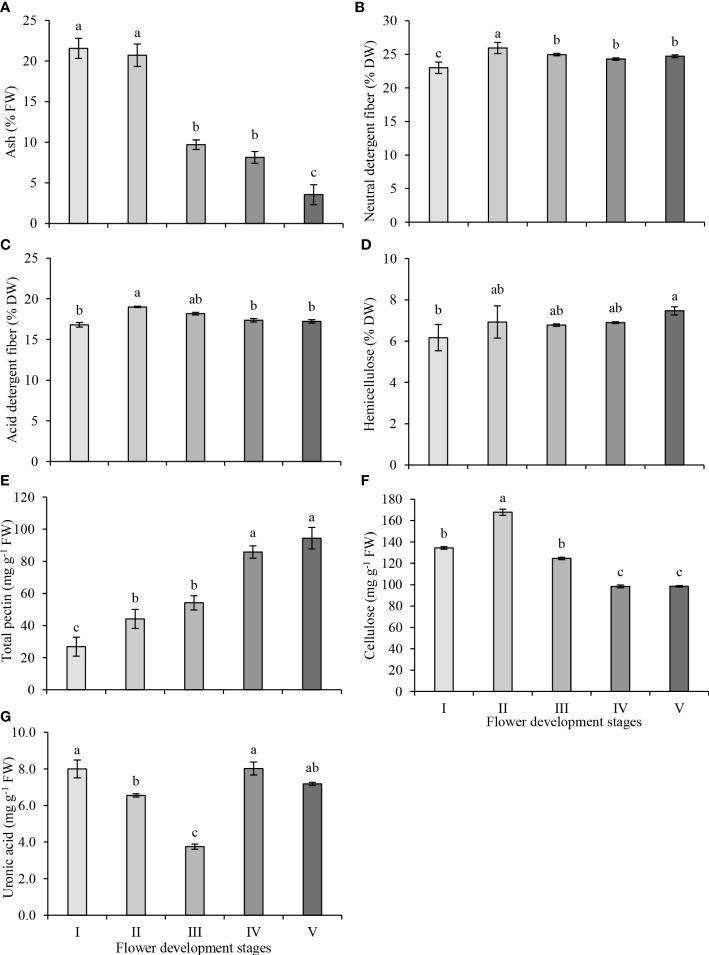
Neutral detergent fiber **(A)**, acid detergent fiber **(B)**, hemicellulose **(C)**, ash **(D)**, total pectin **(E)**, total cellulose **(F)** and uronic acid **(G)** contents in petals at five development stages of oil-bearing rose. Results represent means ± standard deviation. Different letters indicate significant differences at *P*≤ 0.05 level.

The ash content of the petals was highest in stages I and II, and ash content decreased significantly at stage III, remained unchanged at stage IV and decreased further at stage V of flower development. (**Figure 2A**). NDF consists of cellulose, hemicellulose and lignin while ADF consists of cellulose and lignin fractions. The ADF and NDF contents were determined according to the nutritional fiber method, which is defined as the total biomass loss of raw biomass weight after hot water extraction. The changes in NDF and ADF contents were similar. The NDF content increased significantly from stage I to stage II of flower development, decreased at stage III and remained unchanged at the later stages of flower development (**Figure 2B**). While the highest NDF content was observed at stage II (25.94%), the lowest NDF content was observed at stage I (22.99%). The ADF content increased significantly at stage II (19.01%) and decreased significantly at stage IV (**Figure 2C**). The lowest ADF content was observed at stage I (16.82%) of flower development. The difference between the NDF and ADF contents is used to estimate the hemicellulose content of petals. The hemicellulose content of petals remained unchanged between stages I to IV but was higher at stage V compared to stage I of flower development (**Figure 2D**). Consequently, the highest (7.47%) and the lowest (6.17%) hemicellulose contents in petals were observed at stage V and stage I, respectively.

The total pectin content of petals increased steadily as flower development progressed, and total pectin content increased by 63%, 100%, 219% and 248% at stages II, III, IV and V, compared to stage I, respectively (**Figure 2E**). The pectin contents between stages II and III and stages IV and V were not significantly different from each other. The highest and the lowest total pectin contents were observed in stage V (94.4 mg g^−1^) and in stage I (26.9 mg g^−1^), respectively. The cellulose content was higher at early stages (I–III) but was lower at blooming stages (IV and V) of flower development. The cellulose content reached its highest level in stage II (167.8 mg g^−1^), and its level gradually decreased from stage II to IV and remained the same in stages IV and V (98.4 mg g^−1^) (**Figure 2F**). Uronic acids are components of polysaccharides and are present in varying amounts in different plant species. The uronic acid content exhibited marked fluctuations throughout flower development. The uronic acid content decreased from its highest level at stage I (8.0 mg g^−1^) to its lowest level at stage III (3.8 mg g^−1^), then increased at stage IV (8.0 mg g^−1^). Though the uronic acid content slightly decreased at stage V, the amount of this decrease was not statistically significant (**Figure 2G**).

**Figure 3:**
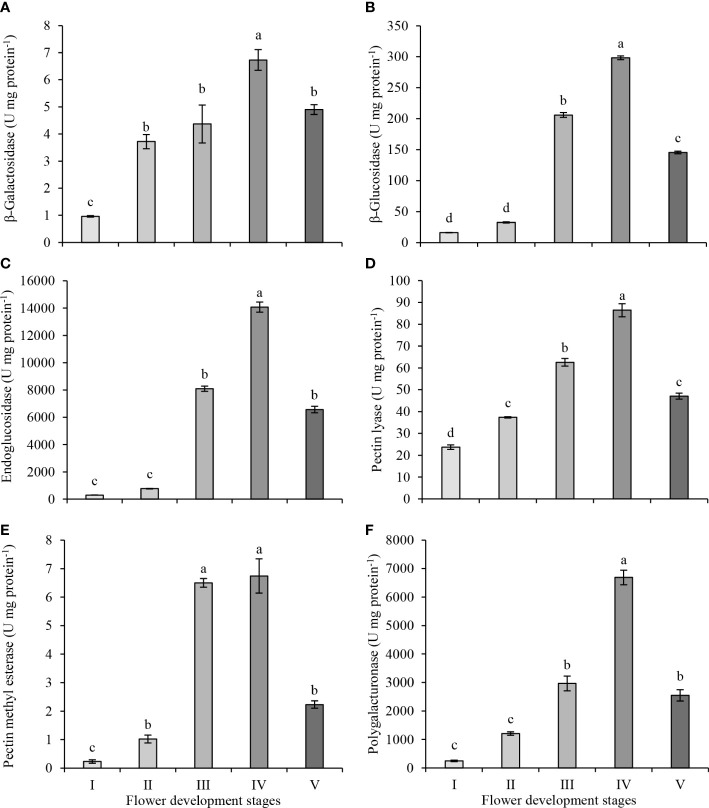
β-galactosidase **(A)**, β-glucosidase **(B)**, endoglucanase **(C)**, pectin lyase **(D)**, pectin methyl esterase **(E)** and polygalacturonase **(F)** enzyme activities in petals of oil-bearing rose sampled at five flower development stages. Results represent means ± standard deviation. Different letters indicate significant differences at *P ≤* 0.05 level.

The plant cell wall is a solid structure consisting of interconnected networks of polysaccharides and proteins. During flower development, the composition and organization of cell wall polysaccharides alter. These changes are mediated by the activities of various cell wall-related enzymes during flower development. The activities of six cell wall-modifying enzymes were investigated in the petals of the oil-bearing rose flowers at five development stages, from green buds to fully opened flowers (**Figure 3**). β-galactosidase activity increased from bud (stage I) to partially opened flower (stage IV) but decreased at full blooming (stage V) (**Figure 3A**). The highest β-galactosidase activity was observed at stage IV (6.73 U mg protein^−1^) and the lowest activity at stage I (0.96 U mg protein^−1^). β-glucosidase activity did not show an increase between stage I and II (16.0 and 32.4 U mg protein^−1^) but significantly increased in stage III (205.9 U mg protein^−1^) and IV (298.3 U mg protein^−1^). At the full blooming stage (V), its activity decreased significantly to 145.5 U mg protein^−1^ (**Figure 3B**). The endoglucanase activities in stages I and II of flower development were 297 and 773 U mg protein^−1^, respectively. The activity in stages III and IV increased to 8097 and 14075 U mg protein^−1^, respectively. Later, the activity decreased to 6566 U mg protein^−1^ at stage V (**Figure 3C**). β-glucosidase and endoglucanase activities started to increase significantly at the stage of intensification of petal coloring and expansion (stage III), and the highest activity in both enzymes was observed in stage IV. Then, β-glucosidase and endoglucanase activities decreased significantly in stage V and fell below those measured at stage III.

Pectin lyase activity increased steadily and reached its highest level at stage IV of flower development (**Figure 3D**). However, at stage V, its activity decreased significantly to 47.1 U mg of protein^−1^, approaching the activity level observed in stage II. The highest pectin lyase activity was in stage IV (86.4 U mg protein^−1^), while the lowest activity was in stage I (23.7 U mg protein^−1^). Pectin methyl esterase and polygalacturonase activities were very low in the first stages of flower development. Pectin methyl esterase activity differed from the other enzymes as it reached its highest activity in stage III (6.5 U mg protein^−1^) and maintained its activity at stage IV (6.7 U mg protein^−1^) of flower development (**Figure 3E**). Later, pectin methyl esterase activity decreased to 2.2 U mg protein^−1^ at stage V. Polygalacturonase activity did not change significantly between stage I and II (**Figure 3F**), but its activity started increasing at stage III (2,966 U mg protein^−1^) and reached its highest activity at stage IV (6,689 U mg protein^−1^) of flower development. Later as with pectin methyl esterase activity, polygalacturonase activity decreased to 2,548 U mg protein^−1^ at blooming (stage V)

**Figure 4:**
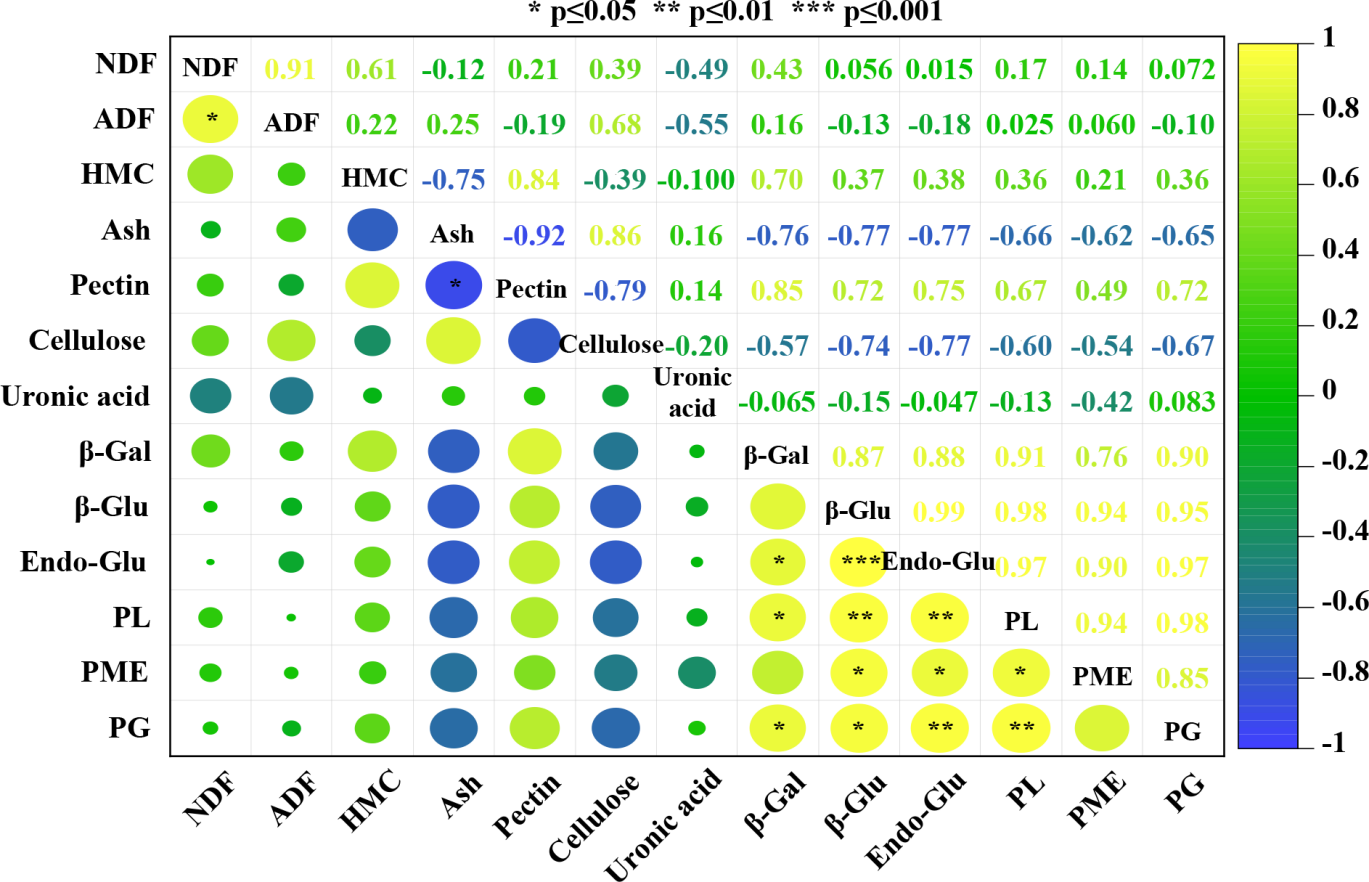
Relationships and correlations between parameters generated by a heat map using mean values. The color scale indicates the intensity of the correlation coefficient values of the different parameters (NDF, neutral detergent fiber; ADF, acid detergent fiber; HMC, hemicellulose; Ash, ash content; Pectin, total pectin; Cellulose; total cellulose; Uronic acid; β-Gal, β-galactosidase; β-Glu, β-glucosidase; Endo-Glu, endoglucanase; PL, pectin lyase; PME, pectin methyl esterase; PG, polygalacturonase). *, ** and *** indicate significance at P≤0.05, P≤0.01 and P≤0.001 levels.

Correlation analysis was carried out to determine the relationships between different cell wall components and cell wall-related enzymes during the flower development of oil-bearing rose. The results of this analysis for five flower development stages of oil-bearing rose are shown in **Figure 4**. Of the 78 correlation coefficients, 14 were positively correlated and one was negatively correlated with each other. Ash content was negatively correlated with total pectin (−0.92) and positively correlated with cellulose (0.86) contents. NDF was positively correlated with ADF (0.91). β-glucosidase, endoglucanase and pectin lyase activities were positively correlated with the other cell wall enzymes. β-galactosidase and pectin methyl esterase activities were positively correlated with pectin lyase and endoglucanase, while polygalacturonase activity was positively correlated with endoglucanase and pectin lyase. Cellulose, hemicellulose and uronic acid contents did not show a significant correlation with any other parameter examined in the study.

**Figure 5:**
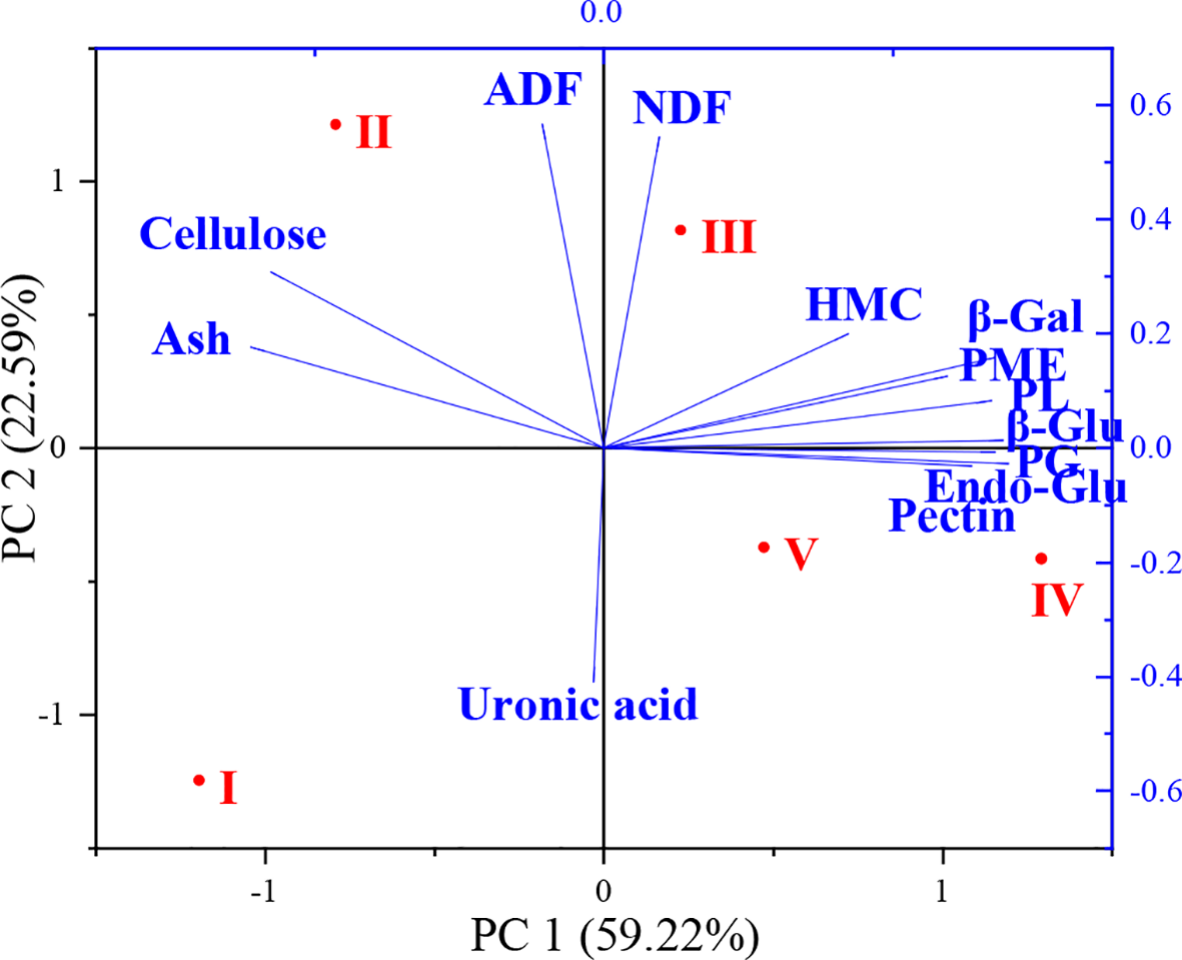
Principal component analysis (PCA) of cell wall composition and related enzyme activities during flower development stages (NDF, neutral detergent fiber; ADF, acid detergent fiber; HMC, hemicellulose; Ash, ash content; Pectin, total pectin; Cellulose; total cellulose; Uronic acid; β-Gal, β-galactosidase; β-Glu, β-glucosidase; Endo-Glu, endoglucanase; PL, pectin lyase; PME, pectin methyl esterase; PG, polygalacturonase).

Relationships between cell wall components, enzyme activities and flower development stages were further analyzed with principal coordinate analysis (PCA) and the results are shown in **Figure 5**. The two principal components (PC) represented 81.81% of the total variation (59.22% and 22.59% for PC1 and PC2, respectively) observed in the study. The different flower development stages were divided into four main clusters. PCA showed that stages IV and V were closely related. The effects of flower development stages on cell wall components and enzyme activities were distributed along the PC1 axis in the order I > II > III > V > IV. Ash, cellulose and ADF content changed significantly at stage II of flower development and clustered on the left side of the PC1 axis. Significant changes occurred in NDF and hemicellulose contents at stage III of flower development. Cell wall enzyme activities were significantly affected in stages III and IV and clustered on the right side of the PC1 axis.

## 4 Discussion

Flower development is related to the structure and alterations of the cell wall due to the activities of cell wall enzymes (Yang et al., 2021), and petal structures also differ among flowering plants. Cell wall structure has been studied in detail in carnation (*Dianthus caryophyllus* L.), sandersonia (*Sandersonia aurantiaca* Hook.), daylily (*Hemerocallis citrina* Baroni) and pigeon orchid (*Dendrobium crumenatum* Sw.) flowers. However, only daylily and pigeon orchid flower development from bud stage to blooming stages and associated changes in cell wall compositions have been reported (de Vetten and Huber, 1990; O’Donoghue et al., 2002; Yap et al., 2008; Yang et al., 2021). These studies showed that the content of cell wall polymers and activities of cell wall-related enzymes differ and these differences are associated with flower development. These studies suggested that flower development may be related to cell wall polymers and metabolism (O’Donoghue, 2006; Yap et al., 2008; Yang et al., 2021), therefore we aimed to determine the cell wall polymer content and cell wall-related enzyme activities of petals of oil-bearing rose from the bud to blooming stages.

Flower development is a complex process that leads to changes in the cell wall structure and polymer contents. In the study, the ash content of developing flowers decreased, and the contents of six cell wall components found in petals differed depending on the flower development stages. NDF is widely used in fiber characterization, as it contains important components of plant cell walls (cellulose, hemicellulose and lignin) and ADF contains cellulose and lignin (Ankom, 2017), thus it is possible to distinguish carbohydrates structurally and non-structurally (Skamarokhova et al., 2020). Hemicelluloses take part in the cross-linking of cellulose in cell walls and contribute to changes in primary cell wall structure (Brummell, 2006). The hemicellulose content of oil-bearing rose petals between the consecutive development stages essentially remained unchanged unlike ADF and NDF contents, which exhibited differences between the development stages. However, the hemicellulose content of pigeon orchid decreased steadily during flower development (Yap et al., 2008). Similarly, ash content decreased during flower development in *Centaurea cyanus* L., whereas the ash content was lower in the bud stage of *Borage officinalis* L. and increased during blooming (Fernandes et al., 2019). Ash content of plants shows high variability depending on physiological and morphological differences as well as genetic and environmental factors (Casler and Boe, 2003; Monti et al., 2008). Since the biomass of flowers can vary greatly due to phenological stage and cultural practices (Monti et al., 2008), the determination of ash content throughout flower development could help determine whether the weight increase of flowers is due to accumulation of cell wall components or due to accumulation of water. Petal area, fresh weight and relative water content increase throughout flower development in oil-bearing rose (Önder et al., 2022), but the very little variation found in ADF, NDF and hemicellulose contents and decreased ash content suggest that the weight increase of petals is mainly caused by accumulation of water and other solutes in oil-bearing rose.

Pectins are major components that mediate remodeling in cell walls (Yang et al., 2021), affect cell adhesion and form the structure of the middle lamella and primary cell wall in dicotyledons (Mohnen, 2008). The total pectin content of oil-bearing rose increased significantly during flower development (**Figure 2E**). Similarly, total pectin content increased steadily during the flower development of pigeon orchid. Pectin content was associated with flowering in *Oncidium* orchids and increased from the blooming development (B stage) to the blooming stage (C stage) (Wang et al., 2008). Cellulose plays an important role in cell wall stiffness by forming cellulose microfibrils that cross-link to the cell wall structure due to its high physical and chemical stability (Liu et al., 2021), therefore cellulose is part of flower development and blooming processes. In oil-bearing rose, the increase in cellulose content at the second stage of flower development and then its reduction at subsequent stages (**Figure 2F**) was similar to the development of goji berry (Liu et al., 2021) and blackberry fruits (Zhang et al., 2019). While the cellulose content showed a decreasing trend after stage II, total pectin content continued to increase during flower development. Similarly, cellulose content started to decrease after blooming, but total pectin content continued to increase until blooming in pigeon orchid (Yap et al., 2008). Uronic acid is the main component of pectin and hemicellulose and is used in flower bud formation and flower development processes (Chakraborty et al., 2022). While the loss of uronic acid continued from stage I to III, uronic acid content increased in stage IV in oil-bearing rose. During olive fruit development, polygalacturonase and pectin lyase enzyme activities increase and uronic acid acid content decrease as maturation progresses, resulting in cell wall degradation and decreased fruit flesh firmness (Diarte et al., 2022). In our study, we have also observed that polygalacturonase and pectin lyase enzyme activities increased until stage IV in which petal expansion becomes visible and blooming starts. Similarly, uronic acid content decreased between stage I and III, but unlike olive fruits, it increased at later stages of flower development and blooming. The significant increase in the uronic acid content after the third developmental stage also implies that the activities of the pectin metabolizing enzymes, which are involved in cell wall remodeling and cell wall breakdown are decreasing.

There are many factors that can regulate modifications of cell wall polymers, including glucosidases and pectinases. Glycosidases play important roles in the breakdown of cell wall polysaccharides and remodeling of the cell wall structure (Minic, 2008). *In vitro* activities of various glucosidases were measured and their activities changed significantly with flower development in oil-bearing rose. Among the cell wall related enzymes analyzed, β-glucosidase (16.03–298.28 U mg protein^−1^), endoglucanase (297.17–14,075.06 U mg protein^−1^), pectin lyase (23.69–86.44 U mg protein^−1^) and polygalacturonase (248.79–6,688.77 U mg protein^−1^) exhibited high activity throughout flower development in oil-bearing rose petals. The other enzymes, β-galactosidase (0.96–6.73 U mg protein^−1^) and pectin methyl esterase (0.23–6.74 U mg protein^−1^), had lower levels of activity. The activity of all cell wall-related enzymes was significantly lower at the full bloom period of flower development (stage V). β-galactosidase, β-glucosidase, endoglucanase, pectin lyase, pectin methyl esterase, and polygalacturonase activities were decreased by 27%, 51%, 53%, 46%, 67% and 62%, at stage V of flower development compared to stage IV, respectively.

β-galactosidase hydrolyzes glycosidic bonds and regulates cell wall flexibility, intercellular communication, and the mobility of enzymes within the cell wall matrix (Brummell and Harpster, 2001; Brummell, 2006). In our study, β-galactosidase activity increased steadily up to stage IV and decreased significantly at stage V. Similarly, β-galactosidase activity increased from the bud stage to the blooming stage and decreased significantly during senescence in sandersonia and daylily flowers (Panavas et al., 1998; O’Donoghue et al., 2002). On the other hand, β-galactosidase activity remained relatively constant until senescence in pigeon orchid (Yap et al., 2008). β-glucosidase is involved in many mechanisms, such as hydrolysis of glycolipids, production of odorant precursors, lignification and catabolism of the cell wall, activation of phytohormones, defense mechanism, flower development and growth (Mishra et al., 2014). In oil-bearing rose flowers, β-glucosidase activity was low during the bud stages (I and II), its activity peaked at stage IV and then decreased at stage V (full bloom stage). Similarly, β-glucosidase activity peaked at the petal separation stage during the flower development of tea (*Camellia sinensis* (L.) Kuntze) and decreased during the full bloom stage (Joshi and Gulati, 2011). β-glucosidase activity increased and the highest increase was observed at the full blooming stages (stages V and VI) of oil-bearing rose (Oka et al., 1999) and paper white (*Narcissus papyraceus* Ker-Gawl.) (Reuveni et al., 1999). However, in pigeon orchid, β-glucosidase activity reached its highest activity at the green bud stage and decreased with the blooming and senescence of flowers (Yap et al., 2008). Endoglucanase plays a role in cell separation, cell wall synthesis, remodeling and degradation (Liu et al., 2011; Jara et al., 2019). During the flower development of oil-bearing rose, endoglucanase activity was the highest at the partially opened flower (IV) and decreased significantly during the full bloom stage (V). Endoglucanase activity has been studied in detail during fruit development and ripening (Jara et al., 2019), but not during flower development. Endoglucanase showed significant activity during softening and ripening of different fruits, such as strawberry (Trainotti et al., 1999), tomato (Rose and Bennett, 1999), avocado (Fischer and Bennett, 1991) and pepper (Harpster et al., 1997).

Pectinases degrade different pectin types. For example, pectin lyase and pectin methyl esterase degrade pectin into short-chain pectin molecules and produce polygalacturonic and galacturonic acids (Yang et al., 2017). Polygalacturonase hydrolyzes pectic substances produced by the pectin methyl esterase, which leads to changes in cell wall properties, such as hardness, elasticity and permeability (Jeong et al., 2015). These events cause loosening of pectins, and structural remodeling and relaxation of the cell wall during cell expansion and cell development (Brummell et al., 2004; Jeong et al., 2015). The continuous increase in pectin lyase activity up to stage IV and a significant decrease in stage V (**Figure 3D**) in oil-bearing rose was similar to daylily flower development (Yang et al., 2021). In addition, the silencing of *SlPL*, which encodes the pectin lyase enzyme in tomatoes, increased fruit firmness and prolonged shelf life, indicating that pectin lyase changes the cell wall structure and affects firmness (Yang et al., 2017). The increase in pectin methylesterase activity was similar to the increase in polygalaturonase activity in oil-bearing rose. Both enzymes had the highest activity at stage IV and their activity dropped significantly at stage V. Pectin methylesterase activity peaked at blooming and then decreased till senescence in sandersonia flowers (O’Donoghue et al., 2002). However, pectin methyl esterase, pectin lyase and polygalacturanse activities in daylily increased throughout flower development and remained high after blooming (Yang et al., 2021). In blackberry fruits, pectin methylesterase and polygalaturonase activity were associated with tissue softening and cell wall relaxation (Zhang et al., 2019), which also occur during the blooming of oil-bearing rose flowers. The activities of some enzymes, such as endoglucosidase and polygalacturonose were very high. It should be noted that *in vitro* enzyme activity may not reflect the enzyme activity in planta due to substrate availability, preferential use of different isoforms, pH and ionic balance of cells and other enzyme activities in plant cell walls (Fry, 2004).

Multivariate analysis methods, such as correlation analysis with heat map and PCA showed the effects of flower developmental stages on cell wall polymers and cell wall enzyme activities. Correlation analysis only found a positive correlation between NDF and ADF, and a negative correlation between pectin and ash content during flower development of oil-bearing rose. However, cell wall-related enzymes showed positive correlations with each other, indicating that their activities during flower development are interlinked with each other. Although their correlation coefficients were not significant, pectin content showed positive and cellulose content negative correlations with the cell wall-related enzymes. PCA showed that stages III, IV and V of flower development were the most physiologically active development stages as revealed by the accumulation of cell wall polymers and enzyme activities.

## 5 Conclusions

Flower development starting from bud to blooming stages in oil-bearing rose is associated with changes in cell wall polymers and cell wall-related enzyme activities. In general, changes in cell wall polymers started to differ significantly at stage II. The petals of oil-bearing rose begin to open in stages III and IV, and, accordingly, the highest cell wall enzyme activities were observed in these stages, indicating that the activities of cell wall-related enzymes are a part of the flowering process. Cell wall structure and its modification is a complex process. To better understand cell wall formation and modification, interactions between non-enzymatic mechanisms and their regulation by cell wall-modifying enzymes may provide further insights into the chemistry and mechanisms of flower development in oil-bearing rose.

## Data availability statement

The original contributions presented in the study are included in the article/supplementary material. Further inquiries can be directed to the corresponding author.

## Author contributions

SÖ and MT designed the experiments. SÖ, DÖ, SE and MM performed the experiments and analyzed the data. SÖ and MT wrote and revised the manuscript. All authors contributed to the article and approved the submitted version.
